# Cell-Free DNA for the Management of Classical Hodgkin Lymphoma

**DOI:** 10.3390/ph14030207

**Published:** 2021-03-02

**Authors:** Vincent Camus, Fabrice Jardin

**Affiliations:** Department of Hematology, Centre Henri Becquerel, 76038 Rouen, France; fabrice.jardin@chb.unicancer.fr

**Keywords:** cell-free DNA, Hodgkin lymphoma, precision medicine, liquid biopsy, circulating tumor DNA

## Abstract

Cell-free DNA (cfDNA) testing, is an emerging “liquid biopsy” tool for noninvasive lymphoma detection, and an increased amount of data are now available to use this technique with accuracy, especially in classical Hodgkin lymphoma (cHL). The advantages of cfDNA include simplicity of repeated blood sample acquisition over time; dynamic, noninvasive, and quantitative analysis; fast turnover time; reasonable cost; and established consistency with results from tumor genomic DNA. cfDNA analysis offers an easy method for genotyping the overall molecular landscape of pediatric and adult cHL and may help in cases of diagnostic difficulties between cHL and other lymphomas. cfDNA levels are correlated with clinical, prognostic, and metabolic features, and may serve as a therapeutic response evaluation tool and as a minimal residual disease (MRD) biomarker in complement to positron emission tomography (PET). Indeed, cfDNA real-time monitoring by fast high-throughput techniques enables the prompt detection of refractory disease or may help to address PET residual hypermetabolic situations during or at the end of treatment. The major recent works presented and described here demonstrated the clinically meaningful applicability of cfDNA testing in diagnostic and theranostic settings, but also in disease risk assessment, therapeutic molecular response, and monitoring of cHL treatments.

## 1. Introduction

### 1.1. Classical Hodgkin’s Lymphoma (cHL) Particularities

Classical Hodgkin’s lymphoma (cHL) is a rare and curable malignancy with an annual incidence representing 10% of new lymphomas and a prevalence of 1% of all cancers in Western countries [1]. Patient outcomes are mostly excellent with multiagent chemotherapy (escalated BEACOPP, ABVD) and modern radiation techniques with a five-year progression-free survival (PFS) rate of 65% to 90% according to advanced versus localized stage disease and standard clinical risk factors [2,3]. Patients achieving an early metabolic rapid response after two cycles of frontline chemotherapy display excellent outcome and are proposed for treatment intensity de-escalation after interim positron emission tomography (PET) assessment [4]. However, approximately one-quarter of patients will display progressive disease or relapse, pinpointing the urgent need to determine the underlying biological processes involved and to select useful biomarkers. At present, no pathognomonic biomarker exists for cHL. To date, the genetic landscape of cHL has been incompletely described because Hodgkin and Reed-Sternberg (HRS) tumor cells are very scarce (0.1–3% of cells in the tissue) [5], hampering molecular biology analyses with techniques that lack sensitivity. 

Standard lymph node removal or tissue biopsy is the recommended procedure for the lymphoma diagnosis and is the routine method for tumor genetic profiling, but this invasive method is associated with numerous caveats: hemorrhage, infections, anesthetic risks, or poor-quality fine-needle biopsy with artifacts or sampling issues [6]. In addition, the rarity of HRS cells augments pathological diagnosis complexity in small biopsy samples. In line with these tumor DNA access difficulties, several teams hypothesized that discovering tumor specific somatic alterations would be more appropriate in the bloodstream, i.e., in cell-free DNA (cfDNA) extracted from plasma. Indeed, various proof-of-concept studies have recently shown that cHL molecular analysis is feasible using cfDNA and highly sensitive methods [7,8,9,10,11]. Despite the scarcity of HRS cells and a typical lower tumor volume, many studies have indicated that success rates for cfDNA detection in cHL are close to those achieved for diffuse large B cell lymphoma (DLBCL). This finding suggests that cHL seems to display a higher trend to release cfDNA than DLBCL. This finding may be related to an increased fraction of tumor cells in apoptosis but also related to the nature of the alterations in the nuclear DNA of reed Sternberg cells [12].

### 1.2. Cell-Free DNA Physiopathology

**Figure 1:**
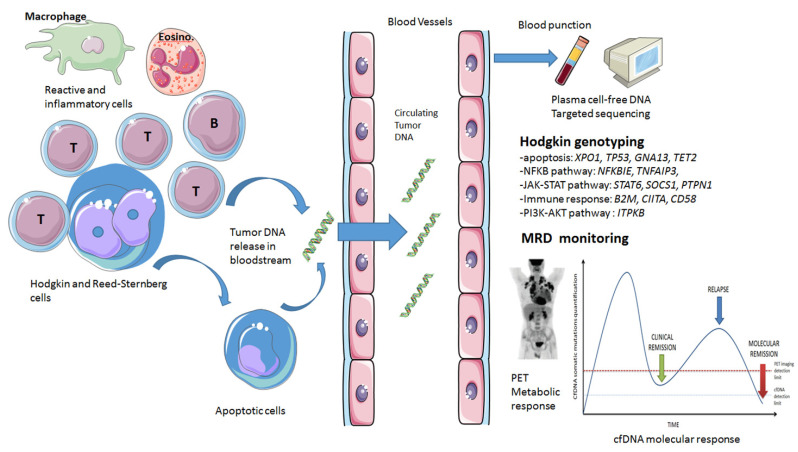
Schematic overview of cell-free DNA in classical Hodgkin lymphoma. Abbreviations: MRD: minimal residual disease.

cfDNA was discovered in the bloodstream several years ago and was first assessed in solid tumors. Previous interesting works in solid tumors settings established that cfDNA fragments may invade distant cells in other tissues, modifying the biology of these cells and contributing to the onset of metastases [13,14]. For cHL, no similar data exist. cfDNA is predominantly freed in plasma by cells in apoptosis and is in addition actively secreted by certain tumor cells or liberated during necrosis phenomenon (see Figure 1). These facts are established by the existing broad spectrum of cfDNA fragment sizes (from 0.150 to several kilobases) and the demonstration of similar genetic profiles in tumor genomic DNA (gDNA) and cfDNA [15,16,17]. Of note, cfDNA exist in healthy subjects and mainly derived from apoptosis of hematopoietic cells. This normal cfDNA is typically detectable in very small quantities in blood [18]. However, these levels increase 15-fold 30 min postexercise and return to normal levels thereafter [19]. The physiological role of cfDNA remains undoubtedly elusive, but a twofold increase in cfDNA levels after the psychosocial stress test and five-fold increase after exhaustive treadmill exercise were recently demonstrated, suggesting that cfDNA is a biomarker of molecular stress [20]. Biological and environmental variables may also modulate cfDNA release, including sex, body composition, age, smoking, exercise, autoimmune disorders, comorbidities, infectious disease, inflammatory conditions, oxidative stress, and pregnancy [21,22]. In addition, cfDNA clearance is also complex and may imply DNase I activity [23], renal clearance [24], and uptake by the liver and spleen followed by macrophage elimination [25]. Several teams measured that the cfDNA half-life in blood is comprised in a range of 16 min to 2.5 h [26] but is largely dependent on several patient settings: healthy subjects vs. cancer patients, before surgery/chemotherapy/radiotherapy or after, and at rest vs. after physical activity [22]. Furthermore, cfDNA clearance is also influenced by binding to cell-surface receptors [27] and several serum proteins [28] (albumin, fibrinogen, prothrombin, and C-reactive protein), the levels of which may considerably vary during the cancer course. All these features complicate biological studies on this topic. It is important to note that cfDNA combined both “normal/non-tumoral” cfDNA and circulating tumor DNA (ctDNA) fragments [18,29], and there is no tool to separate cfDNA arisen from cancer cells and cfDNA liberated by normal cells, which begs the question of background noise and sensitivity of the technologies used in liquid biopsy works to detect somatic variants. Of note, several research teams in solid tumors detected cfDNA in other human fluids, such as cerebrospinal fluid (CSF) in primary central nervous system lymphomas (PCNSLs) with *MYD88* L265P mutation assessment [30,31], urine [32] in the bladder cancer, sputum [33] in lung cancer patients, and uterine lavage fluid in patients with endometrial cancers [34]. In addition, stool DNA may also be valuable in patients with colorectal carcinoma with improved detection rates and a commercially available tool (Cologuard™ assay) [35]. However, these sources of cfDNA seems irrelevant for cHL with no published data to date and no possibility to determine their value for disease burden assessment or genotyping at the time of diagnosis. 

Finally, relevant advantages of plasma cfDNA testing in cHL include: (i) simple venous puncture to obtain sample, (ii) measurable tool which may be performed at any time during patient’s journey (iii) dynamic assessment of clonal evolution, and (iv) less spatial heterogeneity than tissue biopsy genotyping [36,37,38].

### 1.3. Cell-Free DNA Molecular Tools

We know that the optimal way for noninvasive liquid biopsy testing is to extract cfDNA from plasma after blood puncture with nucleic acid preservation tubes (for example, Roche or Streck cfDNA BCT ^®^ [39]). These tubes should then be promptly processed (within 6 h after venous puncture) with consecutive low- and high-speed [40] centrifugations to reduce leukocyte lysis. The intensity of low temperature storage room (−20 °C or −80 °C) remains controversial, but the relevance of leukocyte stabilization tubes is clearly established for easier use [41], especially in multicentric studies. Research teams may experience altered sample quality if they do not satisfy the optimal preanalytical requirements [42]. cfDNA extraction from plasma samples is easy and feasible in most academic laboratories using commercial kits [43,44]. As mentioned above, in cHL, cfDNA comes from rare lymphoma cells and normal cells, thereby necessitating highly sensitive methods for accurate measurement of somatic alterations.

It was previously established that tumor cells in cHL arise from B-cells [45], and clono-specific B-cells are detectable in cHL patients’ blood samples [46]. Normal C-cells and lymphoma cells both expressed B-cell receptor (BCR). BCR variety is a consequence of variable-diversity-joining (VDJ) genes rearrangement during lymphopoiesis. This mechanism provides specific clonotypes, and so each tumor-specific VDJ profile may be considered as a “barcode” for noninvasive tracking of lymphoma in liquid biopsy. Indeed, using ClonoSEQ technology (Adaptive Biotechnologies, Seattle, WA, USA) Oki et al. described a small proof of concept series of seventeen patients, of whom eleven harbored a detectable lymphoma-specific clonotype in tumor biopsies, 8/11 (73%) displayed the same clonotypes in plasma cfDNA, and 33% exhibited the same clonotypes in PBMCs [47]. Using universal VDJ and IdK primers instead of tumor-specific primers, it is possible to detect clonospecific sequences in a single cfDNA sample. This may grant to disclose exhaustive patients’ immunoglobulin repertoire and monitor individual subclones. This ClonoSEQ assay is the sole FDA-cleared minimal residual disease (MRD) tool in lymphomas. Nevertheless, to our knowledge, ClonoSEQ technology results in cHL patients have not been reproduced by other teams, and the sensitivity and specificity of this technique in cHL remain unclear at the moment. Moreover, this technique requires the initial tumor biopsy material for exact assessment of the VDJ profile before tracking it in the blood. In addition, VDJ rearrangements may be unproductive or abortive [48], so the ClonoSEQ method may not work for in this situation, restricting the informativeness of this tool.

In contrast, next-generation sequencing (NGS) gene panel tests may detect concordant potentially “actionable” somatic mutations in the patients’ plasma (cfDNA) and biopsy (gDNA) of lymphoma patients [49] and may contribute to decide appropriate salvage treatments in relapsed/refractory aggressive B-cell lymphoma using new target therapies currently in development. For example, CAncer Personalized Profiling by deep Sequencing (CAPP-seq) is a powerful method for cfDNA measurement that allows deep DNA sequencing and grants an easy detection and quantification of ultralow abundance genetic alterations [50]. CAPP-seq relevance was well described in non-Hodgkin and Hodgkin lymphoma patients. This technology is able to measure disease burden, detect early relapse before radiological progression, perform cell of origin (COO) classification, separate indolent follicular lymphomas and those at risk for high grade transformation, and monitor variants’ clearance in chemosensitive patients versus non-responders patients who display persistent genetic alterations in plasma after treatment [8,51,52,53]. However, such results are only possible at high cost given the elevated number of genes included in the panels and in trained research teams with experienced bioinformaticians able to combine barcoding and unique molecular identifiers (UMIs) with integrated digital error suppression [54]. To date, CAPP-seq is not commercially available. Another report recently assessed cfDNA using real-time PCR in an impressive cohort of 155 pediatric cHL. In this work, the authors showed that baseline cfDNA level is higher in cHL than in healthy subjects, and that higher cfDNA concentration is linked to B-symptoms and inflammatory syndrome. The authors also established that the augmentation of cfDNA concentration after one cycle of chemotherapy led to unfavorable outcome [55].

Finally, digital PCR (dPCR) is a quick, simple and barely costless tool which only needs a small amount of plasma cfDNA. dPCR process dilutes and partitions DNA samples into thousands microcompartments (i.e., microscopic PCR reactors) with each one including a single copy or no copies of the target region [56]. It is then easy to quantify the exact normal or mutated DNA copies number by counting the number of positive compartments with the fluorescent probe corresponding to the wild-type or mutated region. The advantages of dPCR include rapid implementation, the lack of a need for a bioinformatics pipeline and high sensitivity (10^−5^ detection limit), making it a relevant tool when used independently and in addition to NGS for hotspot single-nucleotide variant (SNV) detection, such as *XPO1* E571K (primary mediastinal B-cell lymphoma and cHL) [44]. Indeed, dPCR is based on single-point mutations quantification, and so is not designed to provide the complete molecular landscape of the patient’s lymphoma and, therefore, probably not suitable for molecular response assessment given frequent subclonal evolution. In addition, treatment sensitivity could remove those subclones from cfDNA profiles, so other clones could arise or survive with a false negative dPCR assay. A solution could be to multiplexe dPCR assays to test several hotspot mutations in the same experience, and indeed new dPCR tools can do so (including RainDance^®^ or Biorad^®^) [57,58,59,60]. Furthermore, low amount of cfDNA in some plasma samples impairs the capability to measure molecular response given insufficient haploid genome equivalent quantities. Of note, false positives/background noise issues and technical limits of variant detection are still debated [61,62]. In addition, to date, no published multicenter study of dPCR MRD approaches exists in cHL patients.

Finally, it is important to pinpoint that there is a need for peripheral blood mononuclear cell (PBMC) collection at diagnosis when performing liquid biopsy because hematopoietic clone-derived variants may be detectable in cfDNA samples of both healthy subjects and lymphoma patients and may likely lead to false positive results in cfDNA monitoring experiments [63]. In this situation, gDNA from PBMC may help researchers discriminate polymorphisms from true somatic alterations.

All these molecular biology techniques (summarized in Table 1) are nevertheless interesting and complimentary in the field of cfDNA testing. In this review, we will focus on landmark works in cfDNA assessment in the setting of cHL and we will present clinical practice impact of this tool.

## 2. Genotyping Classical Hodgkin Lymphoma Using cfDNA

### 2.1. Mutational Landscape Obtained by cfDNA Sequencing

A notable NGS study with low-pass sequencing on cfDNA of ten newly-diagnosed localized and advanced stage nodular sclerosis cHL patients revealed genomic imbalances in HRS cells in nine patients at baseline and a rapid clearance after frontline treatment (within a month), revealing cfDNA as a promising tool for molecular response monitoring [11]. In a retrospective proof-of-concept study from our group, including 94 patients with all stages of cHL homogeneously treated with standard frontline chemotherapy, *XPO1* E571K mutations were found using dPCR and NGS experiments in 24.2% of patients. We noted that 29% of all *XPO1 E571K* mutations were only discovered in cfDNA, which may be explained by HRS cell scarcity in cHL. Our group was then able to develop a multigene panel allowing the detection of several somatic alterations of genes involved in the lymphomagenesis of cHL or frequently mutated in this disease. By dPCR and NGS, we found an average of 2.13 mutations per case of cHL; in particular, 30.5% of patients were mutated in the DNA binding domain of STAT6 [9]. However, this panel was only informative for 50% of the patients using cfDNA sources, so we extended it to a nine-gene panel including *SOCS1, XPO1, STAT6, NFKBIE, TNFAIP3, PTPN1, B2M, ITPKB,* and *GNA13.* Our team also led an observational prospective study based on cfDNA testing including 60 consecutive cHL cases treated by frontline ABVD and/or escalated BEACOPP. We observed somatic variants in 42/60 (70%) patients at baseline [10]. However, this gene panel was unable to disclose variants in all of the patients, probably because the panel was too restricted and sensitivity was insufficient to reveal ultra-low abundance subclones which are close to the sequencer limit of detection (variant allele frequency (VAF) 0.1%). The comprehension of cHL biology is growing quickly, and we should include additional genes in next panels. For example, *ATM, KMT2D, TP53, ARID1A*, and *CIITA* are interesting and frequently mutated in cHL. 

In addition, in 2018, Spina et al. reported a major study establishing the genetic panorama of cHL patients using CAPP-seq on plasma-extracted cfDNA. The most commonly mutated genes encompassed *STAT6* (37.5%), *TNFAIP3* (35%), *ITPKB* (27.5%), *GNA13* (18.7%), *B2M* (16.2%), *ATM* (15%), *SPEN* (12.5%), and *XPO1* (11.2%) [8]. The predominance of *STAT6* alterations is a discovery that was never reported in past exome works [64] and clearly reflects the impact of cytokines signaling pathway in cHL [65].

### 2.2. Comparisons between cfDNA and Tumor DNA

In solid tumors [59,60], the similarity rate between variants found in paired tumors and cfDNA samples changed from 88.2% to 64.7% for time intervals of less than three weeks and >3 weeks between venous puncture and tissue biopsy, respectively [60], revealing a sampling time issue. Thompson et al. also demonstrated in lung cancer that increasing the time between tumor and blood collection from < 14 days to >6 months highly reduced the similarity rate [61].

Using CAPP-seq with cfDNA, microdissected HRS-cell enriched areas from biopsies, paired tumor genomic DNA (gDNA) and paired normal gDNA, Spina et al. demonstrated the tumor origin of cfDNA variants depicted in their cHL patients. The similarity (R^2^ = 0.978) of mutational profiles from paired gDNA/cfDNA samples favors the capability of CAPP-seq to precisely detect low burden variants in cfDNA. In our experience, comparability between gDNA and cfDNA profiles with an NGS-limited gene panel is close to 85% [10] at the level variant. Of note, median VAF appears to be higher in cfDNA than in biopsies probable due to the common scarcity of tumor cells in cHL biopsies [10]. In the study by Desch et al. [66] in pediatric cHL patients, the average VAFs were 1.1% for tumor DNA (from whole tissue sections) and 11.1% for cfDNA, but all 30 variants discovered in cfDNA were then confirmed in macrodissected HRS-cell rich regions of paired tumor biopsies, confirming the reliability of cfDNA-obtained mutational profiles. Of note, fresh frozen tissue led to a better concordance rate between genomic DNA and cfDNA (57.1% vs. 66.7% for FFPE tissue) given DNA alterations induced by the FFPE process, particularly for amplicon-based amplification assays [67,68,69]. Nevertheless, this issue could be largely fixed by FFPE DNA repair methods [70] before NGS sequencing. Notwithstanding, tumor subclones are probably dynamically dispersed between various anatomical sites (spatial heterogeneity), which may prevent exhaustive discovery of all possibly existing variants mutations in a unique lymph node resection or fine-needle biopsy. In our opinion, this issue may be surmounted by assessing paired tumor biopsy/plasma cfDNA samples. It now seems established that cfDNA is an excellent mirror of the HRS cell genetic panorama (see Figure 1).

### 2.3. Comparisons between cfDNA Results and cHL Histological Subtypes

In the WHO classification, four distinct cHL subtypes are described [71]: Nodular sclerosis cHL (NSCHL), which is the most common, mixed cellularity (MCCHL), lymphocyte-depleted cHL (LDCHL), and lymphocyte-rich cHL (LRCHL) [72]. Clinical characteristics, overall prognosis, HRS cells phenotype and treatments are similar but the transcriptome and microenvironment show substantial differences. HRS cells typically express *MYC, NOTCH1,* and *IRF4* in all cHL histologic subtypes [73,74].

Nevertheless, gene expression profiling studies demonstrated at the transcriptome level [75,76] that the histologic subtypes of cHL are also biologically distinct. According to Reichel et al., *B2M* mutations are exclusively found in the nodular sclerosis subtype [64]. The work published by Spina et al. [8] confirms these data and indicates that these subtypes are distinct at the genetic level. In particular, NSCHL and EBER-negative cHL are associated with more frequent *STAT6* and *TNFAIP3* cfDNA somatic mutations than other subtypes. Nevertheless, *XPO1* E571K recurrent mutations are detectable in all subtypes and so are not a pathognomonic feature of a particular subtype [7]. Plasma cfDNA concentrations at baseline and genetic profiles from tumor biopsies at diagnosis were also assessed in the 4 cHL subtypes, and no differences were observed in another retrospective study [9].

### 2.4. Potential Interest in the Differential Diagnosis with Other Lymphomas (Gray-Zone, Primary Mediastinal B Cell Lymphoma)

cHL is sometimes hard to diagnose and can therefore be mistaken for several differential diagnoses, including DLBCL, primary mediastinal large B-cell lymphoma (PMBL), anaplastic large cell lymphoma (ALCL), and mediastinal gray-zone lymphoma (MGZL), both of which may display CD30 positivity [71]. In particular, PMBL and NSCHL pathological features are overlapping, so several authors estimated that these two entities are derived from thymic B cells [77,78]. The data demonstrating that the *XPO1* E571K, *STAT6*, and *SOCS1* mutations are frequent PMBL and NSHL but rare in DLBCL [7,8,10,79] support the idea of a shared oncogenic origin between cHL and PMBL. In our opinion, we may use *XPO1* E571K detection by cfDNA analysis to help pathologists to orient between NSCHL, MGZL and DLBCL, especially in the relapse setting if this variant was already present at diagnosis, despite the lack of specificity of this hotspot mutation [79,80,81]. In addition, *STAT6* mutations are easily detectable by cfDNA analysis, are not observed in nodular lymphocyte predominant Hodgkin lymphoma [82] (NLPHL) and are frequent in cHL [8]. Thus, STAT6 mutations may be useful to differentially diagnose these two entities.

## 3. Association of cfDNA Level with Clinical Features

The amount of cfDNA appears to be related to tumor mass and aggressive disease presentation in cHL, making it an interesting prognostic tool starting from diagnosis. Indeed, in a recent prospective study [10], pre-treatment elevated cfDNA concentration was linked to adverse clinical characteristics: age ≥45 years, presence of anemia, albuminemia < 40 g/L, sedimentation rate ≥ 50 mm, stage III– IV disease, lymphocyte count < 0.6 g/L, presence of B symptoms, International Prognostic Index ≥ 3, and elevated lactate dehydrogenase. Patients with mutations in *ITPKB* and *B2M* had more disseminated disease than patients with “negative” plasma. *XPO1* mutated patients were predominantly female with no apparent biological reason for this finding. These results are consistent with those reported in the DLBCL setting [52,53] where higher stage, International Prognostic Index (IPI), and tumor metabolic volume (TMV) are linked to elevated cfDNA levels.

In another study in pediatric cHL patients, augmented cfDNA concentrations were observed in patients aged > 10 year (age ≤ 10: cfDNA range 0–58 ng/mL; age > 10: cfDNA range of 10–1650 ng/mL, *p* = 0.01) [83]. In addition, in the study by Spina et al. [8] *STAT6* alterations were more frequently observed in young patients (<60 years), maybe due to a higher proportion of mixed cellularity and EBER-positive cases in elderly cHL patients.

## 4. cfDNA and Pediatric cHL Specificities

The number of publications concerning circulating DNA in pediatric forms of LH is relatively limited. Simona Primerano et al. [55] determined the pertinence of cfDNA assessment in a series of 155 children with cHL by a Taq-Man-based real-time PCR assay for the *POLR2* with serial venous punctures: at baseline and during frontline chemotherapy. The authors established a control group with 15 pediatric healthy individuals to assess the “normal” (background noise) range of cfDNA in plasma. Median age of the patients was 13.6 years (2.7–19.7) and the authors observed that cfDNA levels were strongly increased in comparison with healty subjects (mean value 112 ng/mL in cHL cases versus 5.5 ng/mL in controls, *p* = 0.002). In this cohort, pre-treatment cfDNA levels were linked to B symptoms presence (*p* = 0.027) and high erythrocyte sedimentation rate (*p* = 0.049). cfDNA kinetics was studied in a subgroup of patients. Interestingly, after one cycle of chemotherapy, an increase in cfDNA was observed in HL with mediastinal bulky disease in 45% of cases. In comparison, only 17% of no bulky disease patients had elevated cfDNA levels after C1. This finding suggests that cfDNA concentration dynamic assessment represent a promising noninvasive tool, especially in bulky mediastinal disease. However, the cfDNA measure performed in this work was not able to distinguish circulating tumor DNA from other sources of circulating DNA, such as myeloid-derived suppressor cells or hematopoiesis cells, during chemotherapy, therefore limiting the meaning of the data.

More recently, Desch and colleagues performed an extensive analysis of tumor circulating DNA in pediatric cHL patients using a hybrid capture-targeted next-generation sequencing assay allowing the detection of single nucleotide variants, insertions/deletions, translocations, and VH-DH-JH rearrangements [66]. Ninety-six pretherapeutic plasma samples from children enrolled in the German cohort of the EuroNet PHL-C2 study were analyzed. Genetic variants were revealed at baseline in cfDNA samples of 72/96 patients (75%), a rate similar to those observed in DLBCL. Variants ranged from 1 to 21 with alleles with frequencies from 0.6 to 42%. Genes implicated in *JAK/STAT*, *NF-kB* and *PI3K* pathways and antigen presentation signaling were most frequently affected.

Sixty percent of all cases displayed *SOCS1* alterations, which is an AID target, and represented the most common variant. The authors add that 118 (29%) of all 399 disclosed somatic mutations affected *SOCS1*. Indeed, *SOCS1* mutations were typically present in multiple copies, deleterious and classified as major mutation events (stops and all indels). Importantly, the authors observed nine translocation breakpoints with various partners, including the *IGH* locus but also *TBLXR1* or *IRF4*. These results indicated that *SOCS1* inactivation is a major and highly selected genetic event in pediatric HL. Consistent with the results obtained in adults, cfDNA levels at baseline were significantly associated with higher TMV. In addition, cfDNA kinetics correlated with the early response assessment by qPET, a quantitative extension of the Deauville scale. Altogether, these studies showed that cfDNA provided a considerable amount of information to genetically characterize HL and monitor therapy response in children with cHL. This technology may offer the possibility of reducing the toxicity of therapies, particularly radiotherapy. However, as in adults, homogenization and validation of analysis techniques are necessary.

## 5. cfDNA as A MRD Biomarker in cHL

cfDNA levels at baseline in newly-diagnosed cHL may predict the patient’s overall outcome (see Figure 1). Indeed, it was recently demonstrated that significantly higher pretreatment median cfDNA quantities at diagnosis were correlated with adverse baseline clinical characteristics, as mentioned above [10]. This finding suggested a link between cfDNA concentration and disease burden and the lymphoma-generated inflammatory syndrome. Although it is not possible to define a reproducible cfDNA concentration threshold predictive of patient survival and their response to treatment, these first results nevertheless indicate that the baseline cfDNA level is a tool for assessing the overall risk of patients in addition to standard clinical, biological, and metabolic imaging prognostic markers. Indeed, in the same series of patients [10], higher MTV at diagnosis is associated with higher cfDNA median VAF. This finding is common in DLBCL [84] and follicular lymphoma [85] settings. Tumor burden in terms of MTV is, therefore, correlated with the quantity of cfDNA released and the overall prognosis of lymphoma. However, this correlation is slight, indicating that several distinct features may confound these two ways of lymphoma burden assessment. The total metabolic tumor surface (TMTS), representing the tumor-host contact, was significantly associated with cfDNA levels in cHL, and the TumBB parameter, representing the dispersion volume of the tumor, was also correlated with cfDNA concentration [86]. It is particularly interesting to understand the association between these new PET parameters and cfDNA because TMTS represents the “battlefront” between the tumor and the microenvironment at the tumor surface where cytolysis and apoptosis occur [87], whereas TumBB is correlated to disease extent and so could be a surrogate marker of Ann Arbor staging. These recent parameters may probably be soon easier to perform because lymphoma imaging will be, in the future, automatically segmented [88]. This will certainly settle the debate whether cfDNA is compliment to PET [86] or is sufficient itself for disease monitoring.

Although cHL patients respond well to treatment with high metabolic CR rates, a very low number of cHL cells can persist and may cause disease to return. This MRD may be measurable with highly sensitive methods.

In a preliminary retrospective work, it was reported that patients with undetectable *XPO1* mutations after frontline chemotherapy had a trend toward a better PFS than those having end of treatment positive cfDNA [7]. We may suggest that the variants clearance in blood is a reliable biomarker of good therapeutic response. In a landmark report, Spina et al. [8] retrospectively analyzed several blood samples in 24 advanced stage cHL patients homogeneously treated with standard ABVD and demonstrated that a 2-log drop in cfDNA between diagnosis and after two chemotherapy courses was linked to complete metabolic response and cure. This threshold was already established in DLBCL [89] and is likely a relevant cutoff to predict disease progression. Therefore, cfDNA assessment adds relevant data to interim PET in determining the therapeutic response. Indeed, cured patients with interim PET positivity had a greater than 2-log reduction in cfDNA. In contrast, relapsed/refractory patients with interim PET negativity had a less than 2-log reduction in cfDNA [8]. In a prospective setting, it was also established that liquid biopsy may provide overall genetic landscape of newly diagnosed and relapsing cHL and may evaluate disease response during and at end of treatment, in compliment to standard PET [10].

In addition, cfDNA may also serve as a tool to identify the clonal evolution of cHL. In the study by Spina et al. [8], a clonal shift between baseline and relapse paired samples was established, which can be tracked by NGS cfDNA analysis. The clonal and subclonal evolution that has been reported by this team is, interestingly, very different depending on whether the patient is receiving treatment by chemotherapy and/or antibody-drug conjugates or treatment by immunotherapy. In the first case, the mutations of the ancestral clone remained detectable in the clone on relapse with new point mutations (*STAT6, GNA13, ITPKB*); in the second case, the ancestral mutations disappeared completely, and the tumor tried to escape immunotherapy by generating completely different mutations. In any case, cfDNA is an excellent tool to follow this MRD and this clonal evolution (see Figure 1).

However, points should be clarified concerning the strategy to be adopted in the event of infraclinical molecular relapse. It is not certain that early retreatment for infraclinical relapse will be positive for patients, will ensure better disease response or that therapeutic intensification in case of a detectable MRD after standard conventional therapy will be associated with better survival. It will therefore certainly take several more years and the establishment of interventional clinical trials based on MRD to be able to integrate this tool into everyday clinical practice.

## 6. Current Challenges and Future Perspectives

Many uncertainties still weigh on our ability to transfer into routine practice the promising data observed with cfDNA in cHL. Remaining issues consist in: (i) lack of sensitivity due to scarcity of HRS cells and low cfDNA quantity in peripheral blood; (ii) limit of detection concerns in plasma cfDNA sequencing background noise (PCR or sequencing errors); (iii) lack of multicenter reproducibility; (iv) lack of uniformity of preanalytical requirements (including storage temperature) and various targeted gene panel; and (v) lack of consensus on the attitude to adopt in the event of evidence of a subclinical relapse, with no localization of the site of the relapse in the event of a positive cfDNA. In addition, NGS requires trained bioinformaticians, robust analysis pipelines and time-consuming dedicated computational validation methods to exclude false-positive artifacts. In our opinion, the central issue lasts the medical significance of “molecular relapse detection” which remains unclear in cHL, instead of acute leukemia settings. Interventional clinical trials based on molecular relapse or positive MRD events are to be imagined to increase the transferability of these recent data into clinical practice.

Finally, it seems important to pinpoint recent research work carried out on alternative liquid biopsy sources to cfDNA plasma. Indeed, platelets have a well-described pivotal role in the immune response to cancer progression [90]. Tumor-educated platelets (TEP) participated in the growth and dissemination of numerous solid tumors and spliced TEP RNA “signatures” (intraplatelet RNA splicing) can grant data on the existence and location of a tumor and also provide molecular genotyping [91]. TEPs were described as promising with several interesting features as compared to standard plasma cfDNA. A great amount of undamaged TEP RNA [92] is accessible in the equivalent of one drop of blood and TEPs grants early pan-cancer detection. Of note, a recent report described a standardized analysis pipeline [93] to enhance reproducibility. Despite the lack of published data in cHL, TEPs will probably be useful in the next future in compliment with other liquid biopsy tools. Finally, extracellular vesicles (EVs) are also an innovative area of research in liquid biopsy. EVs are secreted by normal and tumoral cells, have a size ranging from 30–2000 nm, and have several biological functions, including intracellular communication and tumor microenvironment remodeling [94]. EVs. are not confined to the tumor microenvironment in cancer patients and can be isolated by several yet not standardized methods from blood as well as from other human fluids and so are able to provide good quality tumor RNA and miRNAs [95,96]. For example, *IDH1* somatic alterations in glioblastoma are detectable in mRNA extracted from EVs. using droplet digital PCR sequencing [97]. However, to date there is no published data in cHL regarding EVs. and TEPs and much work remains to be done on these non-invasive exploratory sources of tumor genetic material.

## 7. Conclusions

cfDNA testing is clearly a rising technology in the setting of cHL and is relevant at every step of the patient’s journey. cfDNA is useful to provide an overview of the genomic panorama of the tumor, to assess prognostic stratification and to monitor disease response as a complement to PET. This tool should now be systematically integrated as an endpoint into future clinical trials for cHL at diagnosis and relapse.

## Figures and Tables

**Table 1:** Minimal residual disease (MRD) assessment and “liquid biopsy” methods in patients with classical Hodgkin lymphoma (cHL).

	Plasma Cell-Free DNA		
	Characteristics	Advantages	Disadvantages
cfDNA source	Blood collection: Plasma (Two-step centrifugation process) Standard EDTA or cell-free DNA preservative leukocyte stabilization tubes Well-defined circuit to handle the tubes Less than three years before cfDNA extraction Avoid >2 free-thaw cycles	Easily accessible: Simple venipuncture Extensive data in the literature on cfDNA extracted from plasma Commercial kits for plasma cfDNA extraction	Less than 6 h between venipuncture and storage if EDTA tubes Intensity of low temperature storage room (−20 °C or −80 °C) remains controversial
cfDNA applications	Hodgkin lymphoma genotyping	Non-invasive “liquid biopsy” tool Disease risk assessment; potential interest in the differential diagnosis with other lymphomas (gray-zone, primary mediastinal B cell lymphoma) Potential theranostic interest Excellent mirror of the tumor cell genetic panorama;	Fresh frozen tissue led to a better concordance rate between genomic DNA and cfDNA Tissue biopsy remains “gold standard” for diagnosis
	Minimal residual disease (MRD) monitoring	Dynamic assessment: baseline, during treatment, after treatment, with repeated serial blood samples	Lack of validation to date in large clinical trials No standardized MRD measurement criteria Concerns about background noise and detection limit
	Therapeutic “molecular” response assessment in complement to positron emission tomography (PET)	Add relevant information to PET data, for example in cases of suspected PET false positives or suspected relapse	No standardized technique No multicenter randomized data available for clinical validation
cfDNA tools	High-throughput Ig-VDJ rearrangement sequencing (ClonoSEQ)	Real-time dynamic assessment Commercially available FDA-cleared	Calibration failures (frozen tissue vs. FFPE) Not suitable to tailor targeted therapy No detection of drug-resistant clone during therapy Sensitivity and specificity are unclear
	Panel-directed Next-Generation Sequencing (CAPP-seq and other targeted panels)	Measure disease burden, detect early relapse before radiological progression Monitor variants’ clearance in chemo sensitive patients versus non-responders patients who display persistent genetic alterations in plasma after treatment	No standardized technique Not commercially available high cost due to elevated number of genes included in the panel Need trained research teams with experienced bioinformaticians able to combine barcoding and unique molecular identifiers (UMIs) with integrated digital error suppression
	Droplet digital PCR	Short turnaround tim Low cost Detection of “hotspot” targetable activating mutations Easy serial testing 10^−5^ detection limit	Not commercially available Insufficient data to verify the reproducibility False-positive and detection limit concerns

Abbreviations: Ig: immunoglobulin; CAPP-seq: Cancer Personalized Profiling by deep Sequencing; FDA: Food and Drug administration; PCR: polymerase chain reaction; cfDNA: cell-free DNA.
